# Event and Comment

**Published:** 1914-08-15

**Authors:** 


### DENTAL REGISTER.

**Vol. LXVIII. August 15, 1914. No. 8**

### EVENT AND COMMENT.

NATIONAL DENTAL ASSOCIATION. The Eighteenth Annual Session has just been completed at Rochester, N. Y. This is the first session under the new constitution and it has been anxiously awaited as a test for the new plan. The attendance was large, nearly three thousand dentists were registered and many more visitors. It was expected that in view of the large membership of affiliated societies, that the attendance would be much larger than formerly. In a measure this expectation was verified, but in view of the place of meeting there should nave been a much larger attendance. The Eastern States were not as well represented as would have been the case had the meeting been held in the middle west. It is possible, also, that the members who are not appointed as delegates, particularly in distant places from the place of meeting have felt little obligation to attend as they knew they would be officially represented. If such an impression should become universally prevalent, we fear the attendance will settle down to the delegates and officers only, except from the immediate locality in which the meetings are held.

*Meeting Place:* The sessions were held in municipal buildings constructed by the City of Rochester in a small park about half a mile from the center of city. There are several buildings designed primarily to accommodate an industrial exhibit which is held each fall. The acoustics are, however, admirably adopted for convention purposes and served to accommodate every feature of the Association with ample space and every convenience. The acoustics of the main hall where the sessions were held were not all that one could wish, but the hall was ample in size and free from external noise and diverting incidents. The clinic rooms were ample in size and the arrangements for the clinic good for the most part. Where so many clinics, and on such a variety of subjects are attempted, it is practically impossible to foresee and provide for all needs. The individual methods of clinicians make it not only difficult to provide for their needs, but the spectators do not have a fair chance to see their work. We have thought to solve these difficulties by means of fewer clinics by expert and experienced clinicians, but dentists who attend conventions regularly and frequently get tired of seeing the same clinics so frequently. The old method with all its disadvantages, interests because it encourages the average man to contribute his mite. Both systems prevailed at the National this year and we confess that they both had their faults, but yet we enjoyed them all. The clinic idea seems to be the favorite method of reaching the average dentist, as was evidenced by the way in which they attended the clinics more numerously than they did the lectures and discussions

*Exhibits:* The space set apart for the exhibits by the dealers was one of the most attractively arranged exhibit rooms we have ever seen. One of the large exhibit halls, some 80 by 300 feet in size, was carefully spaced and arranged so that the visitors could pass by every exhibit in an orderly and systematic manner, with plenty of space so that there was no sense of being crowded, or of getting in any ones way. Each exhibit was spaced off by a neat wood frame painted white, with the name of the concern neatly lettered on the top of the frame. The general effect of the whole room was that of a well framed picture. In the center of the room was an ornamental fountain in an artificial lake in which were gold fish, small live ducks, water plants, etc. There were convenient seats for resting, and space for friends to stop and visit as long as they desired. There were over ninety exhibits, practically every well known manufacturer and large dealer in the country was represented. It was, we believe, the most complete exhibit ever held in the country. The credit for the planning and carrying out of this exhibit belongs to Dr. Edward G. Link, of Rochester. In appreciation of their comforts and as an evidence of their entire satisfaction with the excellent work done by Dr. Link, the exhibitors presented him a beautiful diamond scarf pin. It certainly was deserved. His other work for the comfort of the Association brought him election as First Vice-President, which was also well deserved.

*Literary and Clinical Program:* It would be practically impossible for us to record with detail the great work of the convention. When we say that thirty-five papers and addresses, and one hundred and thirty-six clinics were presented in four days, one can get some idea of the magnitude of the meeting from this, the most important aspect of it. To refer to these in anything more than by copying the program would tax our space and our readers patience unnecessarily. Neither can we single out the more important features without unjust discrimination, as there were so many good things said and done that no reporter could hope to do justice to them. It is so big a task that it is like trying to keep track of all the doings of a three-ring circus. All one can fairly do is to give his impressions. The reports made by the scientific research men were probably the most important papers presented. It is promising indeed to know of the amount of excellent work these men have accomplished in a single year, and we are encouraged to hope that in a few more years substantial progress will be assured in the vexing problems undertaken by this committee. It is gratifying to know that the profession is financially backing up the work of this committee. If the work can be kept up a few years as enthusiastically as it has started, there can be little doubt but its future conduct will be adequately provided for. The general comment on the reports indicated that more workers should be provided and that other much needed researches should be begun as soon as possible.

A symposium of papers on dental analgesia and anesthesia and a number of clinics on these subjects evidenced the fact that this is at this time one of the most interesting subjects to the profession. These papers and clinics covered the use of general and local anesthetics by the several materials and methods of application. Nothing especially new was developed in materials used, but a great deal of ingenious technique was presented in the papers and also demonstrated in the clinics. The use of Novocaine for infiltration and the so-called inductive anesthesia was exemplified in a most carefully worked out technique by several clinicians and was adequately presented in the symposium. The use of Nitrous Oxide and Oxygen for local analgesia and general anesthesia was highly commended in the papers and demonstrated in several of the clinics. In the exhibits a number of new and improved appliances for administration were exhibited.

Another subject that seemed to be attracting much attention, judging by the number of clinics given and the interest expressed was in the subject of impression taking and the anatomic articulation of artificial teeth. To some extent this is undoubtedly due to the work done by the tooth and impression compound manufacturers, but we believe the profession is really becoming more interested in the artistic restoration of lost dental organs. It certainly is high time that something better should be produced by our profession, unless we are content to allow this branch of dentistry to go entirely to the "technicker", which would be a backward step indeed.

*Executive Management:* As was to be expected there were some hitches and some confusion in carrying out the details of the official affairs. The most trouble seemed to have been in organizing and conducting the deliberations of the house of delegates. There were delegates who either did not approve or had not mastered the spirit or intent of the new constitution and so believed they could improve it by amendments or by varying its interpretation, and so a deal of talking and adjustment seemed necessary. Then the officers had to be elected and committees appointed by this body and so more trouble and adjusting of affairs had to be done. The President and Secretary will need to be men with wise heads, infinite patience and supreme diplomatic resources to keep things peaceful in this body. -It would be a wise idea for the appointing officer in the various state societies to keep their scrapping members for home consumption, and send to this body only the more judicious and less pugnacious delegates. We don't mean to infer that there was any occasion for a riot alarm on this occasion, but there were enough symptoms to lead one to believe that sufficient provocation with inadequate control in the presiding officer might precipitate some unpleasant scenes and give a chance for unprofessional conduct at times. But after all it is a good place to work off all unpleasant controversies, and it takes such distracting incidents out of the more important public sessions of the convention. This also relieves the general session and the larger mass of the convention from participating in the politics of the association. The large majority is not interested in the politics of the association and does not care to spend the time necessary to learn the details of the executive work of such organizations; and naturally a few men who have given of their time and talents to this kind of work take an interest and even take pride in doing it—such men deserve our special gratitude, but we too often are inclined to censure them, and fail to appreciate their sacrifices. We find ourselves wondering if a smaller delegation would not serve us better and possibly with less friction. We heard the complaint that three States because of the large number of delegates accorded them under the new constitution, dominate by combination the entire convention. While this may be a possibility the result of the election of officers this year does not seem to evidence any combination for that purpose. We have no doubt but we shall by experience make some amendments to the rules of government, but we should give our present constitution an adequate chance to demonstrate its worth rather than to hurry to other experiments.

*Rochester's Hospitality:* We can not close this comment without a few words in commendation of the generous and very satisfactory manner in which we were received into the homes and hearts of the Rochester dentists and citizens. There was none of that extravagant and useless squandering of time and money that has characterized our visits in former years. We were provided with ample and satisfactory entertainment at the excellent hotels of the city and in the homes of the citizens. As we have described above we were provided with ideal facilities for transacting all the work of the convention, and our time was left to our own disposal instead of being used up in excursions and other forms of unnecessary entertainment. Rochester is a most attractive city, especially its suburbs in which are found some of the handsomest parks in the country. A large number of ladies accompanied their husbands to the convention. The social entertainment of the people was largely spent in showing the ladies the city and its many attractions. This they did in a most generous and skillful manner. It was sensible and appropriate from every standpoint, and future conventions might well consider Rochester's methods. We understand that the dentists raised among themselves two thousand dollars for entertaining the convention. In one city a few years ago over six thousand dollars was expended for this purpose. We trust it may not occur again.

*Officers Elected:* Under the new order the officers are elected by the House of Delegates and these officers were elected for the coming year. Don M. Gallie, of Chicago, President; Edward G. Link, of Rochester, First Vice-President; L. P. Dotterer, of Charleston, S. C., Second Vice-President; Dr. Turner, of St. Louis, Third Vice-President; Otto U. King, of Huntington, Ind., Secretary;

H. B. McFadden, of Philadelphia, Treasurer; and the following as trustees: W. E. Boardman, of Boston; H. J. Burkhart, Batavia, N. Y.; Clarence J. Grieves, of Baltimore, Thos. P. Hinman, of Atlanta; M. L. Ward, of Ann Arbor, Mich.; J. P. Buckley, of Chicago; Thos. B. Hartzell, of Minneapolis; Dr. Wherry, of Ogden; C. L. White, of Oklahoma City. The other officers and committees are appointive.

*Printed Proceedings:* The proceedings will be published in the Official Bulletin this year, and a complete list of all committees and appointive officers will be published in due time. The question of a journal for printing all transactions of the Association was again put off until more data can be had and sufficient funds accumulated for successfully launching the venture. The probabilities are that the *Official Bulletin* will gradually assume the functions until it develops into a full fledged monthly journal.

*The President's Address:* It was a comprehensive statement of the work done and to be done by the Association. It contained many suggestions for the betterment of the Profession, notably for the betterment of the educational work in the colleges and for research and post graduate work. It gave a satisfactory statement of the progress made during the year in consolidating and affiliating dental societies in practically every State and Territory. If the newly-elected officers undertake the future work with as much earnestness as the retiring officers, we may look for a most aggressive and successful years work.

*Next Meeting:* The 1915 meeting will be held in San Francisco, in connection with the Panama Exposition. The attendance will undoubtedly be less than at this meeting because of the long travel distance and consequent expense involved. It is, however, due the coast men that the meeting should come to them occasionally, that they may receive some of its benefits. It will be a fine trip for our Eastern men, and, no doubt many will go to see the country. It should be the desire of all who can to go or send such important contributions as shall make the congress a complete success.

*International Congress:* A resolution was adopted inviting the next International Congress for 1919 to meet in New York City, and an earnest effort will be made to secure the meeting.

PREVENTIVE MEDICINE. We are printing a liberal abstract from the address of the President of the American Medical Association, Dr. V. C. Vaughan, delivered at the annual meeting this year. We believe it to be the most comprehensive setting forth of the history of disease development in all ages up to the present. It calls attention in the most emphatic manner to the duty of medical men at the present time. It certainly ought to be read by every young man entering the medical and dental professions, and it may likewise be read by the oldest practitioners with profit and possible satisfaction. It sets forth the true ideal of a professional life, and any one who can grasp the meaning of this address will get a new enthusiasm for his calling that can not fail to materialize in renewed efforts to bring to suffering humanity less of disease and more health. In view of the dentist's obligation for general health it will be the part of wisdom for us to take a larger view of our opportunity as well as of our obligation. The medical profession is awakening rapidly to the conception that what the sick world needs most is better educated physicians and is taking the necessary steps to get wiser and better men into its profession. Are the members of the dental profession to permit selfishness from giving to them similar opportunities for the better education of its members?

